# Hospitals and the Drama

**Published:** 1888-06-16

**Authors:** 


### HOSPITALS AND THE DRAMA.

A most successful dramatic performance was given at the Novelty Theatre, in Great Queen Street, on Tuesday and Wednesday, the 5th and 6th inst., in aid of the Stamford Street Hospital. The pieces selected were Sydney Grundy's charming one-act drama, entitled *In Honour Bound*, in which Mrs. Meadows Taylor, Mrs. George Batten, Mr. Lamb, and Mr. Ivan Watson appeared to great advantage, followed by H. J. Byron's celebrated burlesque of *Little Don Cæsar De Bazan*. The stage management was in the able hands of Mr. Edward Terry, to whom sincere thanks are due for the great success of the performance, which drew a large and fashionable audience on both nights. Mr. Corrie was a most painstaking and able conductor. Mr. Dudley B. Myers, who is now actively associated with Mr. Leonard Broke Willoughby in the development of The Hospitals Association, also took part in the performance.

